# Research Progress in Atopic March

**DOI:** 10.3389/fimmu.2020.01907

**Published:** 2020-08-27

**Authors:** Lan Yang, Jinrong Fu, Yufeng Zhou

**Affiliations:** ^1^Institute of Pediatrics, Children's Hospital of Fudan University, The Shanghai Key Laboratory of Medical Epigenetics, International Co-laboratory of Medical Epigenetics and Metabolism, Ministry of Science and Technology, Institutes of Biomedical Sciences, Fudan University, Shanghai, China; ^2^National Health Commission (NHC) Key Laboratory of Neonatal Diseases, Fudan University, Shanghai, China

**Keywords:** atopic dermatitis, asthma, food allergy, allergic rhinitis, atopic march

## Abstract

The incidence of allergic diseases continues to rise. Cross-sectional and longitudinal studies have indicated that allergic diseases occur in a time-based order: from atopic dermatitis and food allergy in infancy to gradual development into allergic asthma and allergic rhinitis in childhood. This phenomenon is defined as the “atopic march”. Some scholars have suggested that the atopic march does not progress completely in a temporal pattern with genetic and environmental factors. Also, the mechanisms underlying the atopic march are incompletely understood. Nevertheless, the concept of the atopic march provides a new perspective for the mechanistic research, prediction, prevention, and treatment of atopic diseases. Here, we review the epidemiology, related diseases, mechanistic studies, and treatment strategies for the atopic march.

## Introduction

In recent decades, the incidence of allergic diseases has continued to increase, affecting ~20% of the worldwide population, especially children (1). Cross-sectional and longitudinal studies have suggested that allergic diseases occur following a time-based order: from atopic dermatitis (AD) and food allergy in infancy to gradual development into allergic asthma (AA) and allergic rhinitis (AR) in childhood. In terms of anatomic structure, it follows the spatial evolution of skin–gastrointestinal tract–respiratory tract, and this phenomenon is defined as the “atopic march” (2).

Among the allergic diseases mentioned above, some resolve gradually to disappear with age, whereas others continue for many years (3). Some studies have shown that the atopic march does not progress completely in a temporal pattern with genes and the environment (4). Nevertheless, the concept of the atopic march provides a new perspective for the mechanistic research, prediction, prevention, and treatment of allergic diseases.

Here, we review the epidemiology, related diseases, mechanism of action, and treatment strategies of the atopic march.

## Epidemiology of the Atopic March

### AD: The First Manifestation of the Atopic March

AD is a chronic recurrent skin disease. Its clinical manifestations are chronic inflammation of the skin, itching, and an impaired skin barrier. AD affects 3% of adults and ~30% of children, and its prevalence tends to increase with age (5). AD occurs in the early years of life. Some epidemiology studies have shown that 45% of affected children had the condition before 6 months of age, 60% before 1 year of age, and up to 85% before 5 years of age (6, 7).

AD etiology is a combination of various factors involving genes and the environment (8). Once external allergens contact a damaged skin barrier, keratinocytes are stimulated to secrete thymic stromal lymphopoietin (TSLP) and other factors in conjunction with Langerhans cells (LCs) to stimulate T-helper type 2 (Th2) immune responses. Then, the body is stimulated to produce non-specific immunoglobulin (Ig)E (if children are exposed to allergens such as mites for a long time, specific IgE may appear). Subsequently, T cells, eosinophils, macrophages, mast cells, and type 2 innate lymphoid cells (ILC2s) infiltrate to secrete cytokines, resulting in local inflammation of the skin (9). AD patients can be classified into two types based on whether the IgE level is increased: intrinsic (normal IgE and non-allergic) and extrinsic (high IgE level associated with increased disease severity). Studies have shown that extrinsic AD increases the risk of developing the atopic march (10, 11).

### AA and AR: The End Progression of the Atopic March

AA is a common chronic airway disease characterized by the inflammation, hyperresponsiveness, and remodeling of airways (12–15). With modernization and industrialization, AA incidence has increased year by year. This may be because of lifestyle alterations, changes in environmental factors (e.g., increase in indoor dust mites and outdoor pollution), changes in dietary habits, and many other factors. AR involves inflammation of the nasal mucosa (16) and diminishes the quality of life of sufferers (17).

Epidemiologic evidence has revealed a link between AA and AR. A retrospective follow-up study reported the incidence of AR to be higher in AA patients than in non-AA persons (18). In another cohort study, Leynaert et al. demonstrated that 74–81% of AA patients reported AR. Also, they found that AA occurred in 2% of non-AR persons, but in 18.8% of AR patients upon exposure to pollen or animal dander (19).

AR may lead to changes in the function of the lower airways through three main mechanisms. Firstly, stimulation of the nasal mucosa contracts bronchial smooth muscle through the nasal–tracheal reflex. Secondly, various chemical mediators and cytokines released by antigenic stimulation causing nasal mucosa allergy are absorbed into blood, are transported to the lung through circulation, and then act on the trachea and bronchi, causing smooth muscle spasm. Thirdly, nasal inflammatory mediators and secretions are discharged through the nasal passage to the lower airways, resulting in a reduced β-adrenergic receptor functional response (20).

### Epidemiology of the Atopic March: Linking AD With AA or AR

Dharmage et al. found, in infants who have AD within 2 years of age, that the incidence of AA and AR increased significantly during age 6–7 years. In particular, early-onset, persistent, and IgE-positive AD led to a higher risk of developing AA and AR (9). A longitudinal study on a Canadian birth cohort (2,311 children) has shown that AD with sensitization at 1 year of age increased the prevalence of AA and AR at 3 years of age more than 11- and 7-fold, respectively (21). In a recent report from Thailand, 102 children with AD (diagnosed at 1.5 years of age) were reviewed, and subsequently, AR and AA were diagnosed in 61.8 and 29.4%, respectively. Concomitantly, 67% of the AA patients also suffered AR (22). A prospective cohort study (3,124 children aged 1–2 years) reported that, compared with children with no history of AD, those once having AD, particularly moderate-to-severe, early, and persistent AD, were more inclined to develop AA and AR (23).

The discoveries mentioned above strongly support the natural process of the atopic march.

### Roles of Food Allergy

IgE-positive food allergy commonly coexists with AD in early childhood as the earliest manifestation of the atopic march. In 2011, Japanese scholars conducted a retrospective questionnaire survey on freshmen, and they found that AD occurred earlier in those with accompanying food allergy. Also, having food allergy was regarded as the biggest risk factor for the atopic march (24). A family-based cohort study from Chicago revealed that symptomatic food allergy, especially severe or multiple food allergies, was closely related to AA in children aged ≥6 years. Children with food allergy developed AA earlier than those without food allergy (25). A survey of 2,222 infants with AD aged 11.5–25.5 months showed that 64% of children diagnosed with AD within 3 months of birth exhibited an IgE-mediated sensitivity to milk, peanuts, or eggs. Also, in infants <12 months of age, the proportion of infants with sensitivity to eggs, milk, or peanuts increased with AD severity, but this phenomenon was not manifested in children with AD after 1 year of age (26). Among adults with AD, food allergy is relatively rare (27–31). In addition, studies have shown that children sensitive to milk in infancy subsequently exhibited aggravated airway inflammation and increased airway responsiveness to histamine (32, 33). Remarkably, food allergy commonly exists together with AD in infants. Therefore, it is worth exploring whether the link between food allergy and AA or AR is related to AD or is a direct consequence of the food allergy itself.

### EoE: A New Manifestation of the Atopic March?

Eosinophilic esophagitis (EoE) is a chronic esophageal inflammatory disease induced by pollens or food allergens (34). EoE patients are sensitive to allergen avoidance and glucocorticoid therapy. Genome-wide association study (GWAS) data have indicated that EoE shares some susceptible genetic loci with other manifestations of the atopic march, including polymorphisms in the signal transducer and activator of transcription 6 gene (*STAT6*) and *TSLP* (35). In addition, epidemiology studies have demonstrated EoE to be associated with other allergic diseases. For example, Mohammad et al. found that, of 449 EoE patients, the prevalence rates of AR, AA, and AD were 61.9, 39, and 46.1%, respectively, and that up to 21.6% of EoE patients developed these three atopic diseases (36). Another study involving 35,528 people reported that those with IgE-positive food allergy were at a higher risk of EoE (37). A birth cohort study involving 130,435 children determined a positive association between EoE and other allergic manifestations (34).

The studies mentioned above suggest the potential of EoE as the fifth “member” of the atopic march, but this hypothesis is controversial. For example, EoE occurs not only in childhood but also after childhood. In addition, EoE can occur in individuals without a history of atopy. Therefore, larger cohorts are needed to study the epidemiologic relationship of EoE with other manifestations of the atopic march and the mechanisms involved (38).

## Possible Mechanisms Underlying the Atopic March

### Dysfunction of the Skin Barrier

The skin is the foremost barrier for defense against external stimuli, such as pathogens, environmental pollutants, and ultraviolet light. As a component of the innate immune system, the skin has several defensive functions, including microbial, chemical, physical, and immune barrier. These different functions of the skin barrier coordinate with each other to resist external stimuli and maintain skin homeostasis.

Allergens can enter the body through damaged skin to cause sensitization, which is defined as “transcutaneous sensitization” (39). Transcutaneous sensitization can cause AD and, subsequently, AA and AR (5). Studies have shown that epicutaneous disruption induces sensitization after exposure to peanut and egg allergens (40, 41). Spergel et al. demonstrated that repeated cutaneous exposure to egg allergens induced AD-like skin inflammation and AA-like bronchial hyper-responsiveness in a mouse model (41). Emerging studies now suggest that the skin barrier protein filaggrin and epithelial cell-derived cytokines such as TSLP, IL-25, and IL-33 might be related to the progression of the atopic march.

#### Filaggrin

Filaggrin, a barrier protein, has important roles in the integrity of the stratum corneum in terms of structure and composition. Mutations in the filaggrin gene (*FLG*) can impair the barrier function of the skin and induce an allergic response (42, 43). Several studies have shown patients with impaired or reduced levels of filaggrin to be more susceptible to food sensitization (44–46). Moreover, *FLG* mutations increase the risk of early and severe AD and of AA in individuals who have had AD (47–49).

#### Thymic Stromal Lymphopoietin

TSLP is an interleukin (IL)-7-like epithelial cell-derived cytokine which regulates the Th2 response (50). Zhang et al. found that TSLP overexpression in keratinocytes aggravated AA-like airway inflammation in mice subjected to ovalbumin (OVA) sensitization intraperitoneally and OVA challenge intranasally (51). Another *in vivo* study demonstrated that keratinocytic TSLP was essential to induce a Th2 response in the skin and to trigger aeroallergen-challenged AA phenotypes (52). In addition, Noti et al. found that the effect of TSLP was enough to develop experimental EoE-like phenotypes in mice (53). Also, they found that TSLP in skin facilitated food allergy (54).

#### Interleukin-33

IL-33 is derived from epithelial cells and acts on macrophages, ILC2s, Th2 cells, mast cells, and basophils through the suppression of tumorigenicity 2/IL-1 receptor accessory protein heterodimer (*ST2/IL1RL1*) (55–60). Several studies have explored the roles of IL-33 in allergic diseases and found high expressions of IL-33 in the skin or airway epithelial cells in AD or airway inflammation (61–63). Blockade of ST2 expression can alleviate food allergy in peanut- and OVA-challenged models (64, 65).

#### Interleukin-25

IL-25 is also an epithelial cell-derived cytokine (66–68). Kim et al. found that IL-25 inhibited filaggrin expression in the skin and aggravated skin inflammation by coordinating with Th2 cytokines (69). Lee et al. reported OVA/alum-sensitized allergic diarrhea to be inhibited in mice lacking IL-17RB, the receptor of IL-25, whereas IL-25 overexpression in the intestine accelerated the development of allergic diarrhea (70). Kang et al. found that the mRNA expression of IL-25 was upregulated in rat lungs in a TiO_2_-induced model of airway inflammation (71).

**Figure 1:**
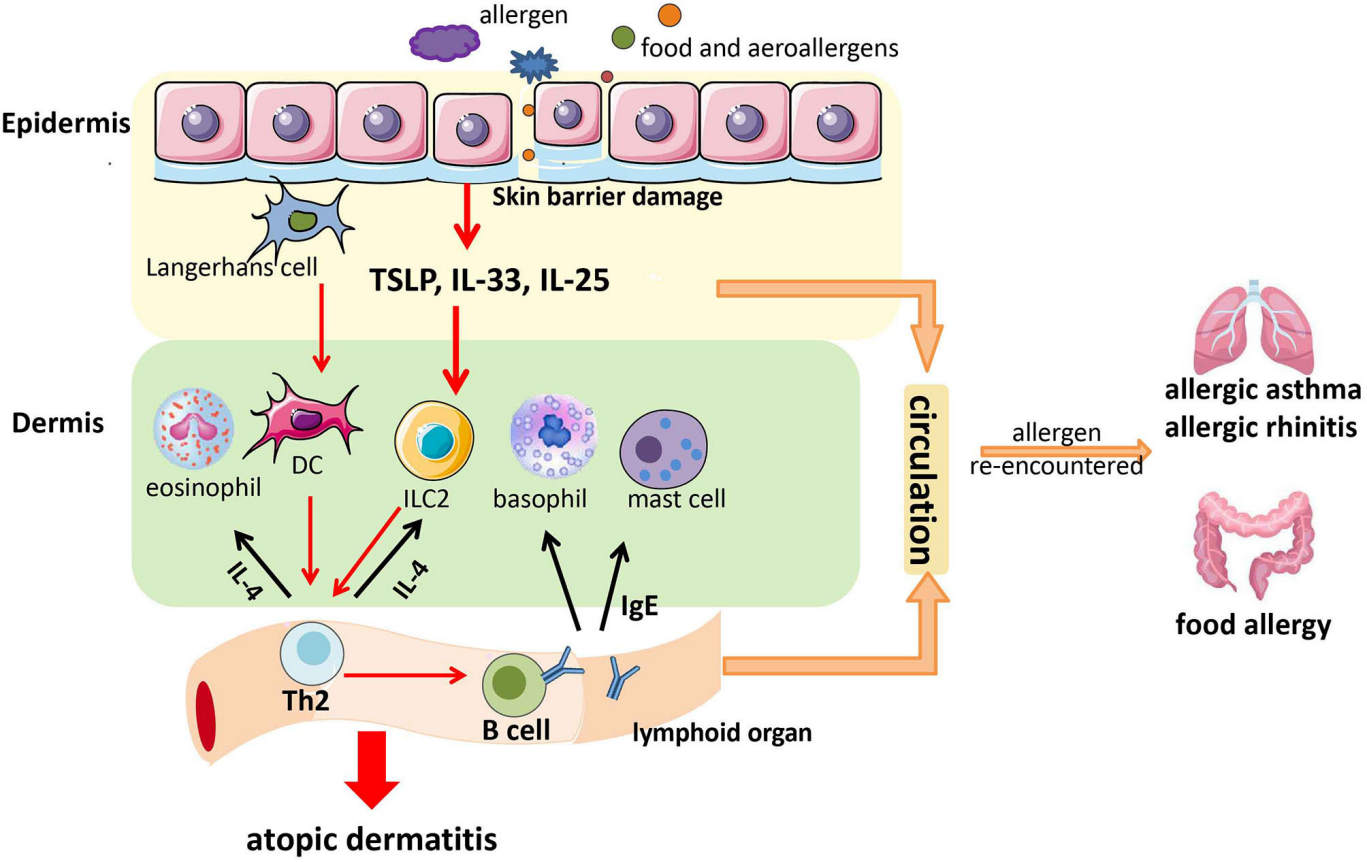
A possible model of the contribution of skin barrier dysfunction to the atopic march. Allergens (including food allergens and aeroallergens) enter the skin through the damaged skin barrier. Then, they stimulate epithelial cells in the skin to release thymic stromal lymphopoietin (TSLP), interleukin (IL)-25, and IL-33. This action activates some immune cells in the dermis (e.g., basophils, mast cells, DCs, eosinophils, and ILC2) to secrete cytokines, followed by the generation of T-helper type 2 (Th2) cells and immunoglobulin E (IgE) production in local lymph nodes. Th2 cells can secrete more type 2 cytokines (e.g., IL-4) to activate more ILC2 and eosinophils, and IgE can act on mast cells and basophils. This positive feedback causes skin inflammation and atopic dermatitis (AD). Furthermore, IgE, Th2 cells, TSLP, IL-25, and IL-33 might enter the digestive tract and respiratory tract through blood circulation to facilitate the development of allergic asthma (AA), allergic rhinitis (AR), and food allergy if allergens are re-encountered.

In conclusion, allergens (including food and aeroallergens) enter the skin through the damaged skin barrier. Then, they stimulate skin epithelial cells to release TSLP, IL-25, and IL-33. This action activates some immune cells in the dermis [e.g., basophils, mast cells, dendritic cells (DCs), eosinophils, ILC2] to secrete cytokines, and subsequently, Th2 cells are generated and IgE production in local lymph nodes occurs. Th2 cells can secrete more type 2 cytokines (e.g., IL-4) to activate more ILC2 and eosinophils, and IgE can act on mast cells and basophils. This positive feedback causes skin inflammation and AD (72). Furthermore, IgE, Th2, TSLP, IL-25, and IL-33 might enter the digestive and respiratory tracts through blood circulation to facilitate the development of AA, AR, and food allergy if allergens are re-encountered (73, 74) (Figure 1). Therefore, skin barrier dysfunction might be a potential mechanism underlying the atopic march.

### Microbiome Alteration

Many microorganisms are colonized in the intestine, skin, and respiratory tract (75) and influence health and disease. Several studies have suggested that microbiome alteration plays roles in atopic diseases locally or peripherally.

Kennedy et al. observed the skin microbiome dysbiosis in early life of AD patients, and they also found that colonization with commensal *Staphylococci* at 2 months was related to a lower risk of AD at 1 year of age (76). Forno et al. and Abrahamsson et al. reported that children who had AD at 6 months (77) and 2 years (78) of age had decreased intestinal microbial diversity at 1 month of life. A study of the KOALA birth cohort demonstrated that infants with *Clostridium difficile* colonization in the gut at 1 month of life were inclined to develop AD and other atopic diseases (79). Azad et al. demonstrated that infants who had a positive skin prick test for food sensitization at 1 year had lower gut microbial richness at 3 months (80). Abrahamsson et al. reported that infants with low diversity in intestinal flora at 1 month of age were inclined to develop AA at school age (81). In addition, Teo et al. determined that microbiome alteration following respiratory infections during infancy might contribute to the development of AA (82).

Moreover, some studies have shown that microbes regulate atopic diseases by secreting metabolites. Furusawa et al. reported that the short-chain fatty acids produced by several intestinal microorganisms induced the proliferation of colonic regulatory T cells (Tregs) and further ameliorated colitis and allergic responses (83). Dysbiosis of *Faecalibacterium prausnitzii*, as observed in AD, was found to reduce the production of butyrate and propionate and further destroyed the intestinal mucosa. Then, some toxins permeated into the circulation and induced a Th2 immune response to facilitate skin inflammation and AD development (84). Johnson et al. found that the polysaccharides derived from *Bacteroides fragilis* induced CD4^+^Foxp3^−^ T cell activation and further prevented AA onset (85).

The studies mentioned above strongly suggest that microbiome alteration may be involved in the atopic march. However, further studies are needed to determine whether microbiome shifts are a cause or a consequence of the atopic march.

### Epigenetic Factors

Epigenetic mechanisms can regulate gene expression and constitute the cause of diseases. Several epigenome-wide studies have revealed DNA methylation in blood to be related to food allergy (86, 87) and AA (88). Recently, Peng et al. undertook DNA methylation analyses on the cohorts of the Generation R Study (343 at mid-childhood and 839 newborns) in the Netherlands and Project Viva (396 at mid-childhood and 232 newborns) in the USA. Meta-analyses linked the differential methylation profiles of the peripheral blood of mid-childhood children with food allergens, environmental/inhalant allergens, and atopic sensitization. Multiple methylation site-related genes were enriched to AA pathways, including eosinophil peroxidase (*EPX*), *IL4*, interleukin 5 receptor A (*IL5RA*), and proteoglycan 2 (*PRG2*). Furthermore, Peng et al. identified several methylation sites of cord blood to be related to allergic phenotypes in mid-childhood and that some methylation sites of cord blood were also present in mid-childhood (89), which suggested a longitudinal time trend.

The findings mentioned above suggest that epigenetics may have roles in allergic diseases. However, these studies show only a correlation between epigenetics and the atopic march. Whether epigenetic change is a cause or a result of the atopic march warrants large and detailed longitudinal studies.

### “Social” Dysfunction of Cells and Molecules

Allergic reactions occur not only in the regions where allergens are in contact directly but also in long-distance, non-contact sites. This may be a systemic reaction of the body, and the mechanisms are incompletely understood. Through literature search, Luo et al. proposed a model of “social events” of cells and molecules to explain the atopic march (90). Epithelial cells, such as epidermal keratinocytes and airway epithelial cells, are the first line of defense against allergen exposure and initiate the inflammatory response by releasing proinflammatory cytokines. Thus, epithelial cells are considered to be key participants in allergic diseases. In this model, Luo and colleagues considered that it is the atopic factors produced by epithelial cells locally, not in the circulation, that drive allergy at different sites and that certain allergens are the irritants that trigger the release of atopic factors at different sites. Zhang et al. reported that TSLP overexpression in keratinocytes induced AD-like symptoms and also aggravated OVA-induced AA manifestations in mice. However, they also found that increased TSLP expression in the skin and, subsequently, peripheral blood was not sufficient to induce lung inflammation (51). The atopic reaction in the lung might be induced by the TSLP derived from the lung epithelia themselves. Therefore, the atopic reaction in the skin and the lung might be the consequence of the “social dysfunction” of homologous epithelia and molecules such as TSLP. Despite its rationality and interpretability, the theory of social events needs sufficient evidence from *in vivo* and *in vitro* studies.

### Interference of Other Predicted Genes

Marenholz et al. performed GWAS on 2,428 cases with AD in infancy and AA in childhood and on 17,034 controls. They identified seven susceptible sites associated with the atopic march: *FLG* [1q21.3], *AP5B1/OVOL1* [11q13.1], *IL4/KIF3A* [5q31.1], *IKZF3* [17q21], *C11orf30/LRRC329, EFHC1* [6p12.3], and *rs99322* [12q21.3] (91).

Bioinformatics analyses by Gupta et al. revealed that the atopic march involved 16 common pathogenic genes: *IL4, IL5, TSLP, RNASE3, IL13, IL10, IGHG4, IFNG, CCL11, FCER2, RNASE2, FOXP3, KCNE4, CD4, IL4R*, and *CCL26* (92). These genes were predicted through large-scale and high-throughput bioinformatics analyses, and their roles in the atopic march need to be determined through further experimentation.

**Figure 2:**
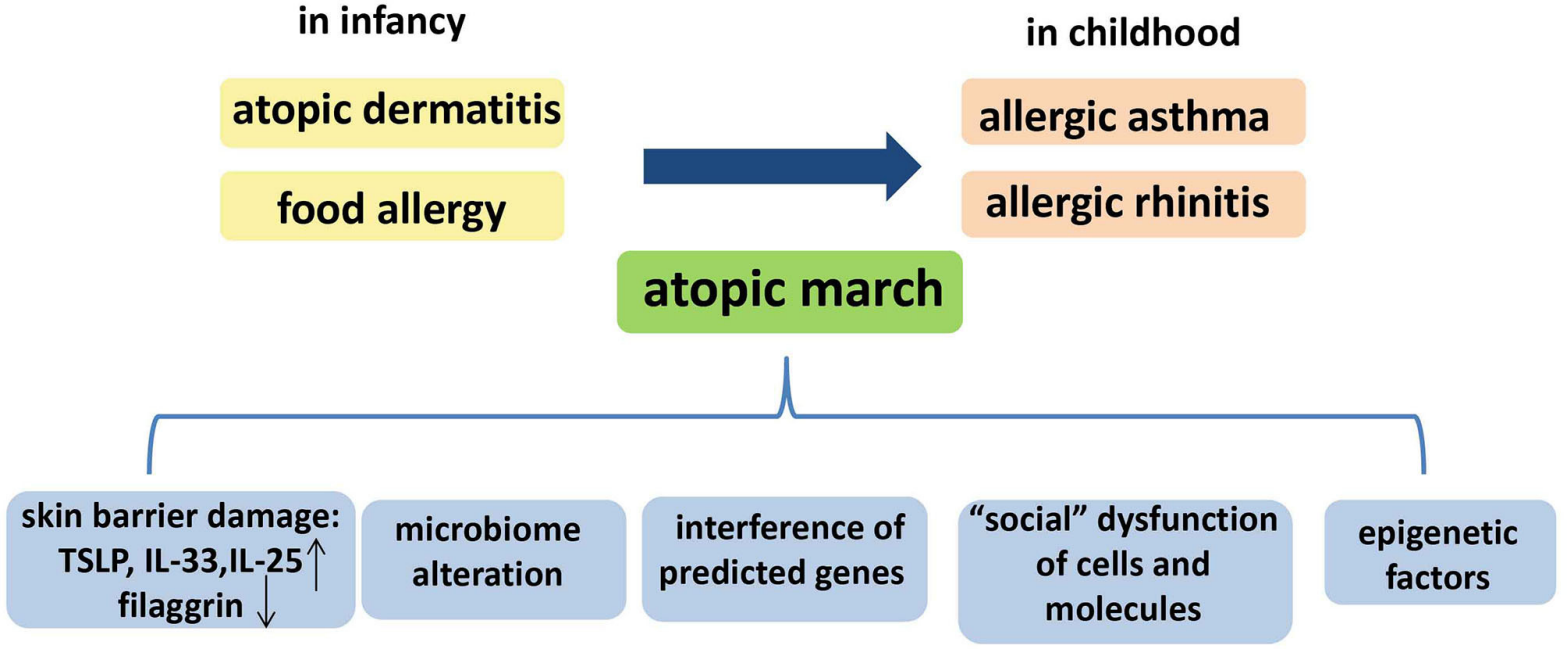
The temporal pattern and the possible mechanisms of the atopic march. The temporal pattern of the atopic march is, in general, from atopic dermatitis (AD) and food allergy in infancy to gradual development into allergic asthma (AA) and allergic rhinitis (AR) in childhood. Several mechanisms could underlie the atopic march: skin barrier damage, microbiome alteration, “social” dysfunction of cells and molecules, epigenetic factors, and interference of other predicted genes.

Summarily, Paller et al. have reviewed the multifactorial etiology of the atopic march, including skin barrier damage, microbiome alteration, and epigenetic factors (93), and we consider that “social” dysfunction of cells and molecules, and the interference of other predicted genes, may also contribute to the atopic march (Figure 2). However, further studies are required to detail the relevant mechanisms.

## Animal Models for Studies on the Atopic March

The modeling process of Leyva-Castillo et al. consisted of two phases. In the first phase, wild-type (WT) BALB/c mice were treated with calcipotriol MC903 plus OVA through epicutaneous sensitization. This led to increased levels of Th2 cytokines, Th17 cytokines, and OVA-specific IgE and IgG1 in serum. In the second phase, MC903-treated (epicutaneous) OVA-sensitized mice underwent intranasal challenge with OVA. These mice exhibited AA-like symptoms with increased mucus secretion, eosinophil infiltration, and expression of Th2 cytokines (52).

In a model established by Han et al., WT BALB/c mice were first treated with OVA plus TSLP *via* the intradermal route (four times within 2 weeks). After 9 days, the mice were challenged by OVA *via* the intranasal route for four consecutive days. Consequently, the mice exhibited increased OVA-specific IgE in serum as well as cellular and eosinophil infiltration in the bronchoalveolar lavage fluid. Histopathology showed severe inflammatory infiltrates in mouse lungs. In addition, periodic acid–Schiff staining showed excessive goblet cell metaplasia and mucus secretion (94).

Moreover, one study showed that epicutaneous exposure to *Aspergillus fumigates* aeroallergens followed by intranasal challenge with *A. fumigates* induced an allergic nasal response in BALB/c mice (95).

In conclusion, the models mentioned above have one similarity: the skin is used as a sensitization site, consistent with the feature that AD is the initial manifestation during the atopic march. These animal models facilitate the studies of the mechanisms underlying the atopic march.

## Refutations of the Atopic March

Despite substantial epidemiologic and experimental evidence, some scholars argued that the prevalence of the atopic march may be overemphasized (96).

First, the methods of the data collection and disease identification initiate one main concern. Considering the cost and time required to make physician diagnoses, allergic disease identification was often based on “yes” or “no” questions. In existing epidemiologic surveys, the diagnosis of AD, AA, and AR simply used “yes” or “no” questionnaires, and some even lacked further physician identification (97–99). In addition, deviations and over-reporting in questionnaire surveys from some individuals led to an overestimation of the disease prevalence (100). Another rebutted criticism of the atopic march is the failure to consider disease heterogeneity or variations. Martinez et al. found that AD patients were at a higher risk of developing transient early AA and persistent AA, not late-onset AA (97). This indicates that the association between AD and AA may be restricted to specific AA subpopulations, not universal. Moreover, some individual-level analyses did not support the typical temporal pattern. At an individual level rather than a large-scale population level, Belgrave et al. demonstrated that only 3.1% of children followed the classical atopic march procession (AD first, followed by AA and then AR) and more than 90% of children with atopic manifestations did not (101).

Unusually, a study from Italy found an evidence of a “reverse” atopic march. The study included 745 children aged 6–9 years with AA only, without a history of food allergy or AD. After a 9-years follow-up, 20% of the children were found to have developed AD (102). In addition, the prevalence of the atopic march differed in distinct countries. Colombian scholars followed up 326 mother–infant pairs in a birth cohort study, and they found that AA was the most common manifestation by 24 months. The prevalence of recurrent AA was 7.1% at 12 months and reached 14.2% at 24 months. However, allergic symptoms induced by milk, egg, or other food allergens were scarce, only 1.8%, and AD was not observed in any cases (103).

Although these studies refute the concept of atopic march to a certain extent, we cannot deny the contribution of the theory of atopic march to the early prevention, diagnosis, and treatment of allergic diseases. Future research on the atopic march should improve the current data collection and disease identification methods, not only relying on “yes” or “no” questionnaires, take disease subtypes into account, and perform the study in an individual level rather than only in a group level.

## Prevention and Treatment Strategies for the Atopic March

Several measures used to prevent and treat allergic diseases are expected to interfere with, delay, and block the natural process of the atopic march.

### Food Interventions

In most studies, breastfeeding for > 6 months has been recommended because it reduces not only the incidence of AD but also of other allergic diseases (104). A 15-years follow-up study of the German Infant Nutritional Intervention (GINI) cohort has shown that, if breastfeeding is not possible, compared with standard cow's milk formula (CMF), the interventional use of partial whey hydrolyzate (pHF-W) formula and extensive casein hydrolyzate (eHF-C) formula in the first 4 months of life has significant preventive effects on AD, and the eHF-C formula also reduced the prevalence of AA and AR (105). However, the mechanisms underlying the preventative effects of hydrolyzed formulas are unknown. In addition, the assessment of the effects of hydrolyzed formulas was based on parental reports of physicians' diagnosis, not on the clinical examinations. These are the criticisms against the use of hydrolyzed formulas for the prevention of allergic conditions.

Moreover, Wickens et al. found that supplementation with *Lactobacillus rhamnosus* for the first 2 years of life reduced the prevalence of AD by about half (106). However, further studies are required to handle the uncertainties about whether other probiotics are equally effective and how probiotics exert their effects on allergic diseases.

Furthermore, the Learning Early About Peanut Allergy (LEAP) trial demonstrated that, compared with children who avoided peanut, sustained peanut consumption, beginning in the first 11 months of life, significantly decreased the prevalence of peanut allergy at 60 months of age in infants with high atopic risk (107). In addition, a large-scale population-based prospective study showed that early introduction of cow's milk protein as a supplement to breastfeeding might promote tolerance, reducing the incidence of IgE-mediated cow's milk protein allergy (108).

### Environmental Prevention

Exposure to several environmental factors is closely related to having allergic diseases. Studies have shown that smoke in the environment increases children's risk of allergic sensitization and AA (109). Therefore, it is strongly recommended that all parents should stop smoking tobacco. Dust mites, pollen, cockroaches, pet fur, and fungi are common allergens, and avoiding exposure to these allergens can reduce the sensitization of children at high risk. However, it has also been proposed that there was no correlation between house dust mite (HDM) exposure and AA (110), and keeping pets (cats or dogs) in the home in the first year after birth reduced the risk of sensitization to multiple allergens during childhood, but it impaired lung function once cat or dog sensitization has occurred, particularly in children with a family history of AA (111). These controversial views are initiating further research to evaluate their relevance.

### Medical Treatment

#### Symptomatic Treatment

Antihistamines are used to relieve itching in AD patients and to prevent skin damage aggravated by scratching. Ketotifen, an H1 antihistamine, significantly lowered AA risk in infants with AD or other pre-asthmatic conditions (112, 113). A double-blind, randomized, placebo-controlled trial showed that compared to placebo, cetirizine significantly reduced the incidence of AA in AD patients sensitized to grass pollen or to HDM (114). However, considering the side effects of antihistamines, a large number of clinical trials are needed to evaluate the security of antihistamines and the effectiveness of interventions in the natural course of allergic diseases.

Glucocorticoid is also an effective anti-inflammatory treatment for allergic diseases, and inhaled glucocorticoids has now become the first-line treatment for AA (115). Although the symptoms of AD and AA can be significantly improved by glucocorticoids, it is prone to relapse after withdrawal. In addition, there are many side effects. Therefore, glucocorticoids should be prescribed with caution by the physicians.

#### Allergen-Specific Immunotherapy

Allergen-specific immunotherapy (ASIT), also known as “desensitization therapy,” can alleviate allergy symptoms for a long time and change the natural course of allergic diseases (116). Several ASIT routes have been documented in preclinical studies, including subcutaneous immunotherapy (SCIT), sublingual immunotherapy (SLIT), epicutaneous immunotherapy (EPIT), and oral immunotherapy (OIT). The recognized mechanism of specific immunotherapy is stimulation of the secretion of IL-10 and transforming growth factor-β from Tregs, promotion of the balance of Th1 cells/Th2 cells, and conversion of IgE to IgG to block the IgE-mediated immune cascade (117, 118). Zhong et al. found that the clinical symptoms and quality of life of AD patients with HDM sensitization could be improved after 2 years of ASIT (119). Besh et al. demonstrated that combining basic therapy with SCIT acquired significantly better results in AA patients compared to basic therapy only (120). Karakoc-Aydiner et al. found that the nasal symptom scores of children with AR were significantly reduced after receiving dust mite allergen vaccine through SCIT or SLIT (121). However, the lack of security greatly limits the development of ASIT. For example, the adverse reactions of SLIT mainly focus on local reactions, such as oromucosal pruritus and gastrointestinal reaction (122). In addition, almost all clinical trials related to OIT are accompanied by one or several serious adverse reactions, such as severe gastrointestinal reactions, systemic allergic reactions, etc. (123). Long-term follow-up of milk OIT patients showed that the complete immune tolerance rate after OIT treatment was only 31% (124, 125). Therefore, further research on ASIT should be directed at the improvement of not only its efficacy but also security.

#### Targeted Therapy

Omalizumab is a human monoclonal antibody against IgE. In 2003, it was approved for the treatment of severe AA in adolescents and adults. Esquivel et al. demonstrated that omalizumab inhibited rhinovirus infections, illnesses, and exacerbations of AA through specific binding to IgE (126). Dupilumab is a human IgG4 monoclonal antibody against IL-4 receptor subunit alpha (IL-4Rα), and it can inhibit IL-4 and IL-13 signaling pathways by interacting with IL-4Rα (127). Dupilumab has been approved by the US Food and Drug Administration to treat infants with moderate-to-severe AD with poor results from conventional treatment (128). Tezepelumab (AMG 157) is a monoclonal antibody (G2λ) against TSLP. In one clinical trial, tezepelumab treatment for 5–12 weeks blunted inhaled allergen-induced AA attacks (129). Of note is that these targeted therapy medicines are only licensed for use in certain allergic diseases. Although the off-label uses or adjunct to treatment for numerous allergic conditions have acquired encouraging results, their potential efficacy still needs to be evaluated through clinical trials.

## Biomarkers of the Atopic March

Although there are no reliable biomarkers to identify subjects with high risk of atopic march, Davidson et al. have proposed some recommendations recently for future research to explore biomarkers, which would provide some possibilities to examine the atopic march.

The relevant proposals are as follows: (1) to look at the protein, RNA, and lipid signatures in infants before and after AD using multi-omics approaches; (2) to analyze transcriptomics, proteomics, metabolomics, and the cell types of infant blood sequentially; (3) to perform sequential immune profiling of the blood, including serology, cytokine profiles, and the evolution of specific B and T cells; (4) to investigate the microbiomes in the skin and gut from birth; and (5) to consider potential maternal delivery effects for atopy (130).

## Conclusion

The global increase of atopic diseases greatly lowers the quality of life. The theory of atopic march facilitates our understandings of the pathophysiology of atopic diseases and further promotes the early detection, prevention, and treatment of children at risk of allergic diseases. Future studies on atopic march would be directed at the following points. Firstly, the methods for data collection should be improved and disease heterogeneity or variations should be considered when performing substantial epidemiologic surveys. Secondly, more detailed and logical mechanisms, including genetic and environmental aspects, should be explored to account for the temporal pattern, which would pave the way for novel approaches for the prevention and timely early treatment of the clinical manifestations, ultimately reducing the allergy burden.

## Author Contributions

LY contributed to collection of references and manuscript preparation. JF and YZ contributed to manuscript modifications. All authors contributed to the article and approved the submitted version.

## Conflict of Interest

The authors declare that the research was conducted in the absence of any commercial or financial relationships that could be construed as a potential conflict of interest.
